# Cytoplasmic ERβ Localization and NTS/NTSR1 Expression in Uterine Leiomyosarcoma: An Immunohistochemical Insight

**DOI:** 10.3390/reports9030199

**Published:** 2026-06-24

**Authors:** Yurena Rodríguez, Francisco Montes de Oca, Idaira Dorta, Ricardo Reyes, Aixa R. Bello

**Affiliations:** 1 Departamento de Bioquímica, Microbiología, Biología Celular y Genética, Área de Biología Celular, Facultad de Ciencias, Sección de Biología, Universidad de La Laguna, 38200 La Laguna, Spain; 2 Hospital Quirónsalud Tenerife, 38006 Santa Cruz de Tenerife, Spain; 3 Instituto de Enfermedades Tropicales y Salud Pública de Canarias (IUETSP), 38200 La Laguna, Spain; 4 Instituto de Tecnologías Biomédicas (ITB), 38206 La Laguna, Spain

**Keywords:** leiomyosarcoma, ERβ, neurotensin, NTSR1, myometrium, immunohistochemistry, biomarkers

## Abstract

Uterine leiomyosarcoma (LMS) is a rare and aggressive malignancy with diagnostic challenges, particularly in cases with overlapping histological features with atypical leiomyoma or smooth muscle tumors of uncertain malignant potential. We report a comparative immunohistochemical analysis of LMS, leiomyoma, and adjacent myometrium obtained from a 40-year-old woman with discordant pathological diagnoses. LMS tissue showed increased Ki67 and NTS/NTSR1 immunoreactivity together with a distinctive cytoplasmic localization of estrogen receptor beta (ERβ), in contrast to the predominantly nuclear localization observed in leiomyoma and normal myometrium. Importantly, focal areas within adjacent morphologically non-neoplastic myometrium exhibited an immunophenotype resembling LMS, including cytoplasmic ERβ localization and increased Ki67 and NTS/NTSR1 expression. These observations suggest a potential association between ERβ subcellular localization, NTS/NTSR1 signaling, and molecular alterations occurring during uterine smooth muscle tumorigenesis. However, given the single-case nature of this report, these findings should be considered exploratory and require validation in larger studies. The diagnostic message conveyed by these images may assist in the interpretation of diagnostically challenging cases and provide a basis for future investigation.

Uterine smooth muscle tumors encompass a spectrum ranging from benign leiomyomas to malignant leiomyosarcomas (LMS), with intermediate entities such as leiomyoma with bizarre nuclei and smooth muscle tumors of uncertain malignant potential. Distinguishing between these entities may be challenging, particularly in cases with overlapping histological features, and reliable biomarkers remain limited [1,2,3,4,5,6].

Recent advances highlight the need for additional biomarkers to improve diagnostic accuracy and prognostic assessment [1,2,3,4,5,6]. Among these, steroid hormone receptors and proliferation/apoptosis markers such as Ki67 and p53 have been widely investigated [7]. The neurotensin (NTS)/neurotensin receptor 1 (NTSR1) system has also emerged as a relevant pathway in tumorigenesis due to its role in proliferation, migration, and angiogenesis [8,9].

In this study we report a comparative immunohistochemical analysis of LMS, leiomyoma, and adjacent myometrium from a single patient, focusing on ERβ subcellular localization and NTS/NTSR1 expression to identify potential markers of malignant transformation.

A 40-year-old woman presented with abnormal uterine bleeding and was diagnosed with a 5 cm intramural-submucosal leiomyoma. Following myomectomy, histopathological evaluation revealed a tumor with increased mitotic activity, cytological atypia, and infiltrative features (Figure 1a,b), leading to discordant diagnoses including LMS and atypical leiomyoma. Due to this uncertainty, a hysterectomy was subsequently performed, revealing multiple leiomyomas. Following total hysterectomy, the patient remained clinically stable, with no evidence of local recurrence, distant metastasis, or other disease-related complications during a 2-year follow-up period.

Immunohistochemistry was performed on formalin-fixed, paraffin-embedded tissue sections of LMS, leiomyoma, and adjacent myometrium. After deparaffinization and rehydration, antigen retrieval was carried out in 0.1 M citrate buffer (pH 6.0) for 5 min at 90 °C. Non-specific binding was blocked with 1% fetal bovine serum (FBS) in TBS. Sections were incubated overnight at 4 °C with primary antibodies against Ki67, p53, ERα, ERβ, progesterone receptor (PR), neurotensin (NTS), and neurotensin receptor 1 (NTSR1). After incubation with the primary antibodies, sections were incubated with the corresponding peroxidase-conjugated secondary antibodies, and immunoreactivity was detected using either DAB or chloro-naphthol. Detailed antibody information is summarized in Table S1. Appropriate positive and negative controls were included in each staining run. Immunoreactivity was evaluated semi-quantitatively by visual assessment of staining intensity and distribution of positive cells. Staining was classified as absent (−), weak (+), moderate (++), strong (+++), or very strong (++++) based on the overall staining pattern observed in each tissue type. LMS tissue showed increased Ki67 and moderate p53 expression (Figure 2a,c), together with strong NTS and NTSR1 immunoreactivity (Figure 2g,i). Immunoreactive cells for both ERα and ERβ (Table 1) were detected. ERα was consistently localized in the nucleus, whereas ERβ displayed a predominantly cytoplasmic distribution in LMS cells (Figure 2e).

In contrast, leiomyoma samples showed minimal proliferative activity and weak NTS/NTSR1 expression, with ERβ restricted to the nucleus. Importantly, focal areas within the adjacent myometrium exhibited an immunophenotype resembling LMS, including increased Ki67 and p53 positivity (Figure 2b,d), NTS/NTSR1 expression (Figure 2h,j), and cytoplasmic ERβ localization (Figure 2f). A comparative immunohistochemical profile of LMS, LM and adjacent myometrium is summarized in Table 1.

The diagnostic message conveyed by these images is twofold. First, the cytoplasmic localization of ERβ in LMS contrasts with its nuclear localization in benign tissue, suggesting altered receptor signaling, possibly related to tumorigenesis and tumor progression, as has been suggested in other gynecological malignancies such as serous ovarian cancer and vulvar squamous cell carcinoma [10,11]. Second, the presence of similar alterations in morphologically non-neoplastic myometrium is compatible with the possibility that molecular changes may occur before overt histological evidence of malignancy becomes apparent. In this sense, internal positive controls showed preserved nuclear ERβ localization in adjacent benign smooth muscle cells, supporting the specificity of the observed cytoplasmic staining pattern and arguing against a generalized staining artifact. These observations are consistent with previous reports suggesting a potential biological continuum in uterine smooth muscle tumorigenesis [3,6,7]. However, alternative explanations, including microscopic tumor extension, field effects, reactive changes, or other local tissue alterations, cannot be excluded and should be considered when interpreting these findings.

The observation of focal myometrial areas displaying an LMS-like immunophenotype despite the absence of overt histopathological abnormalities may be of particular biological and diagnostic interest. As illustrated in Figure 1c, the adjacent myometrium showed no overt histopathological abnormalities on routine H&E examination. The focal areas displaying the LMS-like immunophenotype were identified in serial sections of this adjacent myometrium. Although the significance of this finding cannot be determined from a single case, it raises the possibility that molecular alterations associated with tumor development may extend beyond morphologically recognizable tumor tissue. Alternatively, these findings could reflect local field effects, microscopic tumor extension, or reactive changes within the surrounding myometrium. From a diagnostic perspective, such observations may be relevant in borderline or otherwise challenging smooth muscle lesions, where conventional histopathological criteria alone do not always provide unequivocal classification. Further studies are required to determine the prevalence and biological significance of these alterations in uterine smooth muscle tumors.

In addition, the NTS/NTSR1 pathway has been implicated in tumor proliferation, migration, and angiogenesis in different cancer types [9,12,13,14]. Although its role in uterine LMS remains incompletely understood, previous studies, including work from our group, have reported NTS and NTSR1 expression in uterine leiomyoma and LMS, suggesting a potential involvement of this signaling pathway in tumor biology [13,15]. In the present case, the increased expression observed in LMS and in adjacent myometrial areas with atypical immunophenotype raises the possibility that this signaling axis may be involved in early tumorigenic events. However, further studies are required to clarify its biological significance and potential clinical relevance in uterine LMS. The combined evaluation of ERβ subcellular localization and NTS/NTSR1 expression is not proposed as a substitute for established histopathological criteria or routinely used immunohistochemical markers such as Ki67 and p53, which remain fundamental for the diagnosis of uterine smooth muscle tumors. Rather, these markers may provide complementary biological information, particularly in diagnostically challenging lesions such as STUMP, atypical leiomyomas, or other borderline smooth muscle tumors where conventional morphological and immunohistochemical findings may not always be conclusive. In addition, the altered immunophenotype observed in focal areas of morphologically unremarkable myometrium suggests that these markers may help identify biological alterations that are not readily apparent on routine histopathological examination.

In conclusion, the images presented illustrate distinct immunohistochemical patterns associated with uterine LMS, including ERβ cytoplasmic localization and NTS/NTSR1 activation, and highlight a potentially relevant immunophenotypic pattern that may be associated with uterine smooth muscle tumorigenesis. Given the single-case nature of this report, these observations should be considered exploratory and hypothesis-generating, requiring validation in larger studies before any diagnostic or biological significance can be established.

## Figures and Tables

**Figure 1:**
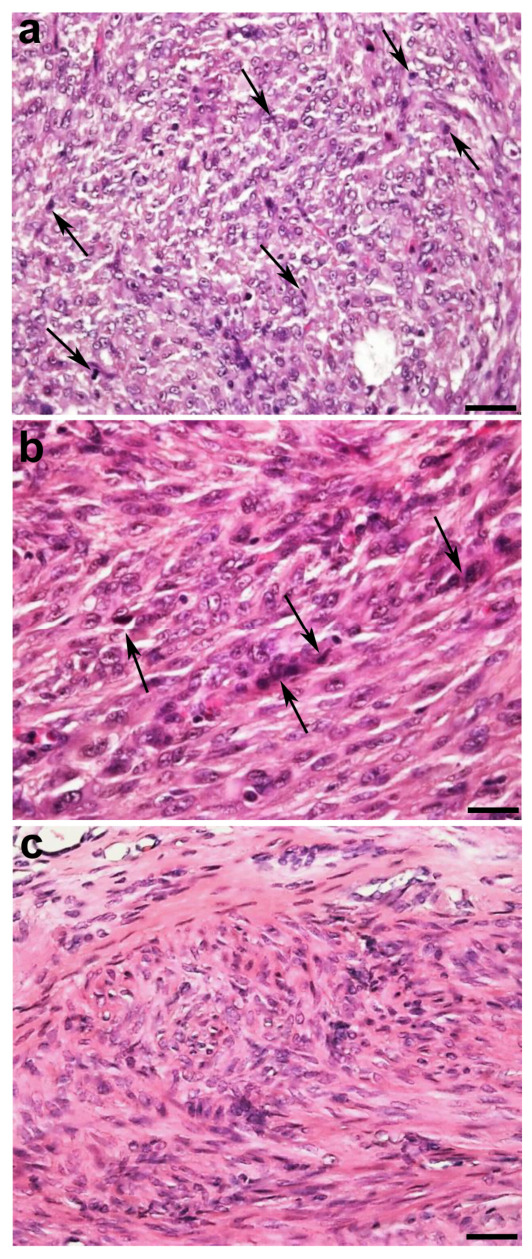
Histopathological features supporting the diagnosis of uterine leiomyosarcoma in a diagnostically challenging smooth muscle tumor. Hematoxylin–eosin staining shows increased mitotic activity (arrows) (**a**) and marked cytological atypia (arrows) (**b**), findings that initially raised suspicion of malignancy despite subsequent discordant pathological interpretations. (**c**) Adjacent myometrium showing preserved smooth muscle architecture and no overt histopathological abnormalities on routine H&E examination. Scale bars: 50 µm (**a**,**c**), 30 µm (**b**).

**Figure 2:**
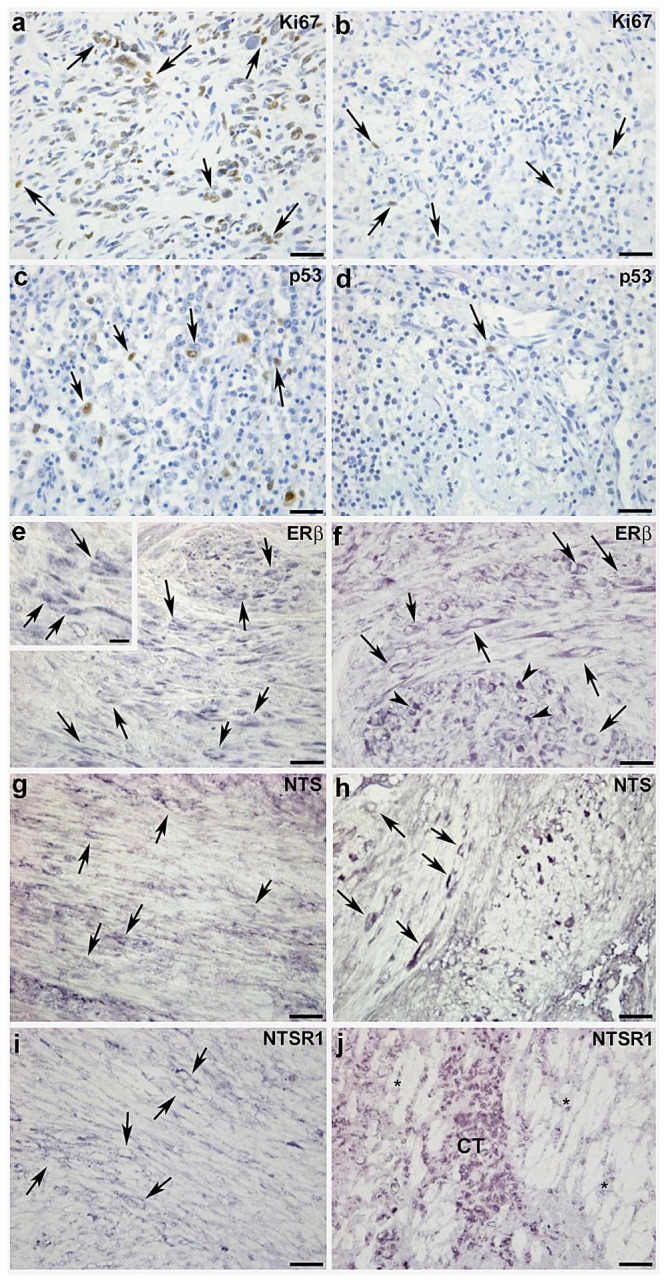
Immunohistochemical images revealing early molecular alterations associated with uterine leiomyosarcoma. LMS cells show increased Ki67 and p53 nuclear immunoreactivity (**a**,**c**), strong NTS and NTSR1 expression (**g**,**i**), and a distinctive cytoplasmic localization of ERβ (**e**). Importantly, focal areas within adjacent morphologically non-neoplastic myometrium display a similar immunophenotype, including increased Ki67 and p53 expression (**b**,**d**), cytoplasmic ERβ localization ((**f**), arrows), and increased NTS/NTSR1 immunoreactivity (**h**,**j**), suggesting molecular alterations preceding overt histological malignancy. Arrowheads indicate myometrial cells with preserved nuclear ERβ localization. Asterisks in image (**j**) indicate NTSR1 immunoreactivity in smooth muscle areas. CT: Connective tissue. (**a**–**d**) Ki67 and p53 immunostaining visualized with DAB and hematoxylin counterstain. (**e**–**j**) ERβ, NTS and NTSR1 immunostaining visualized using chloro-naphthol as chromogenic substrate. Scale bar: 30 µm.

**Table 1:** Comparative semi-quantitative immunohistochemical scoring of LMS, leiomyoma and myometrium based on visual assessment of staining intensity and distribution of positive cells: −, absent; +, weak; ++, moderate; +++, strong; ++++, very strong. +/++ indicates heterogeneous staining intensity between weak and moderate levels.

Biomarker	Myometrium	Leiomyoma	Leiomyosarcoma
PR	+	+++	++++
ERα	++	++	++
ERβ *	++	++	++++
NTS	+	++	++++
NTSR1	+	++	++++
p53	−	+	+/++
Ki67	+	+	++

* ERβ showed predominantly nuclear localization in myometrium and leiomyoma, whereas a predominantly cytoplasmic localization was observed in leiomyosarcoma.

## Data Availability

The original contributions presented in this work are included in the article. Further inquiries can be directed to the corresponding authors.

## Supplementary Materials

The following supporting information can be downloaded at: https://www.mdpi.com/article/10.3390/reports9030199/s1, Table S1: Antibodies and immunohistochemical procedures used in this study.
