# Supplementary material for: Cytoplasmic ERβ Localization and NTS/NTSR1 Expression in Uterine Leiomyosarcoma: An Immunohistochemical Insight
Source: Reports (MDPI). 2026 Jun 24;9(3):199. doi: 10.3390/reports9030199 (PMC13398042; doi:10.3390/reports9030199)
Supplement: Supplementary file 1 [file reports-09-00199-s001.zip › reports-4357422-supplementary.pdf]

Table S1. Antibodies and immunohistochemical procedures used in this study. Information includes antibody source, host species, clone (when applicable), dilution, secondary antibody, incubation conditions, and chromogenic detection system.

Antigen	Antibody	Source	Dilution	Secondary antibody	Dilution	Chromogen
NTS	Rabbit polyclonal	Peninsula Laboratories	1:200	Goat anti-rabbit POD	1:500	Chloro-naphthol
NTSR1	Goat polyclonal	Santa Cruz Biotechnology	1:200	Rabbit anti-goat POD	1:500	Chloro-naphthol
ERα	Rabbit polyclonal	Santa Cruz Biotechnology	1:300	Goat anti-rabbit POD	1:500	DAB
ERβ	Rabbit polyclonal	Santa Cruz Biotechnology	1:300	Goat anti-rabbit POD	1:500	Chloro-naphthol
PR	Rabbit polyclonal	Santa Cruz Biotechnology	1:300	Goat anti-rabbit POD	1:500	DAB
Ki67	Mouse monoclonal (MIB-1; GA626)	Dako	1:100	Goat anti-mouse POD	1:300	DAB
p53	Mouse monoclonal (DO-7; M7001)	Dako	1:100	Goat anti-mouse POD	1:300	DAB
